# Changes in the European Union definition for endocrine disruptors: how many molecules remain a cause for concern? The example of crop protection products used in agriculture in France in the six last decades

**DOI:** 10.3389/fpubh.2023.1343047

**Published:** 2024-01-16

**Authors:** Cloé Roger, Adèle Paul, Emmanuel Fort, Céline Lamouroux, Areejit Samal, Johan Spinosi, Barbara Charbotel

**Affiliations:** ^1^University Lyon, Umrestte UMR T 9405 (University Claude Bernard Lyon 1 and Gustave Eiffel), Lyon, France; ^2^CRPPE de Lyon, Hospices Civils de Lyon, Hôpital Lyon Sud, Lyon, France; ^3^The Institute of Mathematical Sciences, A CI of Homi Bhabha National Institute, Chennai, India; ^4^Santé Publique France, French National Public Health Agency, Paris, France

**Keywords:** crop protection products, endocrine disrupting, occupational exposure, endocrine target organs, occupational medicine

## Abstract

**Background:**

The endocrine-disrupting effects of phytopharmaceutical active substances (PAS) on human health are a public health concern. The CIPATOX-PE database, created in 2018, listed the PAS authorized in France between 1961 and 2014 presenting endocrine-disrupting effects for humans according to data from official international organizations. Since the creation of CIPATOX-PE, European regulations have changed, and new initiatives identifying substances with endocrine-disrupting effects have been implemented and new PAS have been licensed.

**Objectives:**

The study aimed to update the CIPATOX-PE database by considering new 2018 European endocrine-disrupting effect identification criteria as well as the new PAS authorized on the market in France since 2015.

**Methods:**

The endocrine-disrupting effect assessment of PAS from five international governmental and non-governmental initiatives was reviewed, and levels of evidence were retained by these initiatives for eighteen endocrine target organs.

**Results:**

The synthesis of the identified endocrine-disrupting effects allowed to assign an endocrine-disrupting effect level of concern for 241 PAS among 980 authorized in France between 1961 and 2021. Thus, according to the updated CIPATOX-PE data, 44 PAS (18.3%) had an endocrine-disrupting effect classified as “high concern,” 133 PAS (55.2%) “concern,” and 64 PAS (26.6%) “unknown effect” in the current state of knowledge. In the study, 42 PAS with an endocrine-disrupting effect of “high concern” are similarly classified in CIPATOX-PE-2018 and 2021, and 2 new PAS were identified as having an endocrine-disrupting effect of “high concern” in the update, and both were previously classified with an endocrine-disrupting effect of “concern” in CIPATOX-PE-2018. Finally, a PAS was identified as having an endocrine-disrupting effect of “high concern” in CIPATOX-PE-2018 but is now classified as a PAS not investigated for endocrine-disrupting effects in CIPATOX-PE-2021. The endocrine target organs associated with the largest number of PAS with an endocrine-disrupting effect of “high concern” is the reproductive system with 31 PAS. This is followed by the thyroid with 25 PAS and the hypothalamic–pituitary axis (excluding the gonadotropic axis) with 5 PAS.

**Discussion:**

The proposed endocrine-disrupting effect indicator, which is not a regulatory classification, can be used as an epidemiological tool for occupational risks and surveillance.

## 1 Introduction

### 1.1 Endocrine disruptors effects

The concept of endocrine disruptor is a recent one, first emerging in 1991 at the Wingspread conference (1). During the conference, scientists shared their findings and concerns about the health consequences of chemicals that can interact with the endocrine system. Long before the emergence of this concept, numerous examples of endocrine disruption had already been observed.

Tributyltin is a good example of the endocrine-disrupting effects identified in the environment (2). Tributyltin was used in paints in the 1970s to limit the binding of algae and shellfish to boat hulls. The death of thousands of female whelks and nucelles led to its banning in France in 1982. In fact, female nucelles developed a penis (“imposex”) which obstructed the expulsion of their eggs, leading to their death.

In the area of human effects, we can cite the example of diethylstilbestrol (3), a synthetic estrogen with a greater estrogenic effect than estradiol. Since 1947, diethylstilbestrol had been widely dispensed to pregnant women to prevent miscarriage. The rationale for prescribing this treatment assumed that estradiol levels were falling before childbirth. Despite the publication of numerous studies demonstrating the ineffectiveness of diethylstilbestrol in the early 1950s, the treatment was still prescribed to millions of pregnant women. In 1970, clear-cell adenocarcinomas of the vagina were observed in young women who had been exposed *in utero* to diethylstilbestrol even though this cancer was rare in this age group (2, 4). This led to a ban on prescribing diethylstilbestrol in the 1970s in several countries. Subsequently, other effects of this synthetic estrogen were observed, such as a slightly higher risk of breast cancer in women exposed to diethylstilbestrol, but above all effects on their offspring. Indeed, in addition to an increased incidence of vaginal cancer in the daughters of exposed women, reproductive and fertility disorders (premature menopause and higher rates of miscarriage) have been described (4). Sons are also subject to these effects. They present genital malformations such as cryptorchidism. Effects have also been described in the grandchildren of women exposed to diethylstilbestrol. They are said to be at increased risk of male genital malformations and possible attention deficit hyperactivity disorder (ADHD) (3).

The World Health Organization proposed a definition of endocrine disruptors as early as 2002 (5). Thus, the definition adopted was “exogenous substance or mixture which alters the functions of the endocrine system and consequently induces harmful effects on the health of an intact organism (or) of its descendants.”

Endocrine disruptors interfere with the action of hormones, disrupting processes from fetal development to adulthood. They exert their effects at multiple levels by binding to hormone receptors. For example, they can modify the number of hormone receptors in different cell types and the transport, synthesis, concentration, and elimination of circulating hormones. Hormones are molecules secreted into the bloodstream by endocrine cells. They act on target cells—which have specific, highly affinity receptors—and thus finely regulate hormone-dependent organs and tissues. Hormones influence development and physiological processes (2). A single endocrine disruptor may link to several types of hormone receptors, thus exerting different effects (4).

There are periods of developmental vulnerability during which exposure to endocrine disruptors is critical. These include the *in utero* period, the post-natal period, and the adolescence period. These periods are highly sensitive to and dependent on the influence of hormones. The action of hormones during development has different effects depending on these periods, and these effects can persist throughout life. Thus, an organism does not suffer the same effects when contact with an endocrine disruptor occurs *in utero*, before or after puberty. During fetal development, all organs are forming, and endocrine feedback mechanisms are not yet mature. They may still depend on those of the mother, who may themselves be subject to endocrine disruption (3). Even in adulthood, and therefore outside these periods of vulnerability, endocrine disruptors exercise effects by interacting with hormone receptors (e.g., diethylstilbestrol and the occurrence of mammary tumors in women exposed to this substance in adulthood) (4). Some endocrine disruptors can induce epigenetic changes that can have transgenerational effects (4, 6). Exposure to endocrine disruptors *in utero* or in the neonatal period can cause disorders that may only appear in adulthood (6).

One characteristic of the hormonal system is its non-monotonic dose–response curves. With regard to the effect of endocrine disruptors, this implies that greater effects can be observed at lower doses of endocrine disruptors than at higher doses. Thus, exposure to levels below the authorized limit values does not exclude the absence of health consequences (4).

Finally, the cocktail effect is a particular characteristic of endocrine disruptors (7). This means that endocrine disruptors can have additive, synergistic, or subtractive effects. They sometimes result from the addition of the effects of several compounds present at low doses and acting on the same biological pathways.

### 1.2 The CIPATOX-PE project

CIPATOX-PE was created in 2018 to document the knowledge of the endocrine disruptors (ED) effects on health of phytopharmaceutical active substances (PAS) (8) The study aimed to identify the endocrine-disrupting effects for all PAS that had a marketing authorization (MA) in France between 1961 and 2014. These data on health effects came from five governmental organizations: “Endocrine Disruptor Screening Program” (EDSP) (9), “Endocrine Disruptor Strategy” (EDS) (10), “Joint Meetings on Pesticides Residues” (JMPR) (11), “the Strategy for Identification of Endocrine Disruptors and Evaluation of their Cumulative Risks” (European Food Safety Authority, EFSA 2016) (12), and “Classification, Labelling and Packaging” (CLP) (13).

CIPATOX-PE-2018 had identified the endocrine-disrupting effects of PAS according to the WHO definition but also according to the European “interim” regulatory criteria for identifying substances with endocrine-disrupting effects, which included, in particular, certain carcinogenic and reprotoxic classifications of substances based on CLP data (14, 15). These “interim” criteria, issued pending the adoption of specific and validated criteria, were therefore the existing guidelines in effect when CIPATOX-PE-2018 was created. This has led to the inclusion of endocrine gland toxic effects (EGT) in addition to specific endocrine-disrupting effects.

Nowadays, the WHO definition is widely accepted by the scientific community (3) and is used as a reference for many initiatives to identify substances with endocrine-disrupting effects. For instance, the European Commission (EC) adopted in October 2018 a new definition for the determination of endocrine-disrupting properties, aligned with the WHO definition (16) and repealing therefore the “interim” criteria. Thus, if a PAS is identified as having an endocrine-disrupting effect, the regulations provide that it cannot be authorized on the market (16).

The objective of this study was to update the CIPATOX-PE database by considering these new European endocrine-disrupting effect identification criteria as well as the new PAS authorized on the market in France since 2015. The update is also motivated by the constant improvement of the literature on endocrine-disrupting effects, with the availability of new endocrine-disrupting effect identification initiatives, some of them being non-governmental.

## 2 Materials and methods

### 2.1 CIPA database and list of PAS

The list of PAS reviewed for endocrine-disrupting effects in the CIPATOX-PE database is based on the French CIPA database (Compilation des Index Phytosanitaires Acta) (17). In 2021, it indexed the PAS that had obtained marketing authorization in France from 1961 to 2021.

The Chemical Abstracts Service (CAS) numbers corresponding to the PAS, which are not listed in the ACTA indexes, were searched in a second step. The CAS numbers previously filled in for each PAS in CIPATOX-PE-2018 were conserved.

As microorganisms (MOs) are not assessed for endocrine-disrupting effects, 57 MOs listed as PAS were not considered in this study. Indeed, according to the regulations (15), crop protection products include MOs such as fungi and viruses (14). However, as part of the 2018 guidance (18) published by the European Chemicals Agency (ECHA) and the European Food Safety Authority (EFSA) for the implementation of the current European criteria, the term “substance” was used in reference to “chemical substances.” Therefore, microbiological active substances are currently considered by EFSA to be out of the scope of the regulation.

### 2.2 Endocrine-disrupting effect identification initiatives

In accordance with the WHO definition and the new European regulation definition (3, 16), CLP data are no longer included in CIPATOX-PE-2021. Similarly, endocrine gland toxicity (EGT) does not follow the same toxicological rules as endocrine disruption and is no longer in line with the current European criteria.

In order to obtain data on endocrine-disrupting effects of PAS, as was done in CIPATOX-PE-2018, a search for initiatives identifying these effects was conducted. As the WHO definition is now consensual (3), non-governmental endocrine-disrupting effect identification initiatives based on this definition were considered. The intention was to allow a complementary approach as the scientific resources used may differ.

**Figure 1:**
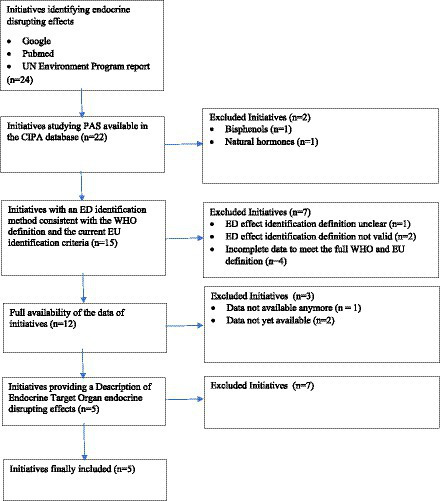
Flow chart describing the identification of initiatives of interest for the update of CIPATOX-PE-2021.

The first step was to inventory international initiatives aiming at identifying substances with endocrine-disrupting effects. The report published by the United Nations Environment Program (UNEP) in 2018 (19) provides an inventory of initiatives identifying endocrine-disrupting effects up to March 2017. A complementary search was carried out—on the search engine “PubMed” (20) and generalist “Google” (21)—for initiatives that have been carried out after publication or that have not been mentioned by UNEP. Twenty-four initiatives were identified by this research. These initiatives were then analyzed according to the four following criteria (Figure 1).

#### 2.2.1 Initiatives studying the PAS listed in the CIPA database

Two initiatives were excluded because they did not include the PAS listed in the CIPA database, with one of them addressing bisphenols [Kemikalieinspektionen, Swedish Chemicals Agency (KEMI)] (22) and natural and synthetic hormones for the other (Endocrine-Disrupting Chemicals in the Australian Riverine Environment Land & Water Australia) (23).

#### 2.2.2 Initiatives with an endocrine-disrupting effect identification method consistent with the WHO definition and the current EU identification criteria

Seven initiatives were excluded because the definition of an endocrine-disrupting effect was not clearly determined (chemicals purported to be endocrine disrupters, Institute for Environment and Health, 2005) (24), the definition used was obsolete (Pesticide Action Network International list) (25), “Publication of the list of pesticide products containing a potentially endocrine-disrupting substance, Ministry of ecological transition” (26) or the data seemed incomplete to match the full WHO and EU definition, e.g., Endocrine Disruption exchange, 2018 (27), EDCs Databank (28), Endocrine Disruptor Knowledge Base et Estrogenic Activity Database—Food and Drug Administration (29, 30), and Endocrine Disruption Screening Program for the 21st Century Dashboard (31).

#### 2.2.3 Availability of initiative data

Two initiatives were not yet available at the time of this study (Endocrine Active Substances Information System [EASIS 2.0 (32)]), Extended Tasks on Endocrine Disruption [EXTEND, 2016 (33)] and were therefore excluded.

#### 2.2.4 Initiatives providing a characterization of endocrine-disrupting effects on endocrine target organs

In light of this criterion, seven other initiatives were excluded as follows:

– The Endocrine Disruptor Lists, published by The Danish Environmental Protection Agency (34)
– List of substances of interest as regard to a potential endocrine activity from the French Agency for Food, Environmental, and Occupational Health & Safety (ANSES) (35)
– European Union Impact Assessment on Criteria to Identify Endocrine Disruptors (36)
– International Panel on Chemical Pollution (IPCP) (19)
– Trade Union Priority List for REACH Authorization (37),
– Substitute it Now! List (38)
The ECHA list of substances of very high concern (SVHC) (39)

For two of the seven initiatives not selected—although there were data on endocrine-disrupting effects—their descriptions, levels of evidence, the target organism, and endocrine organs studied were not systematically reported, leading to their exclusion (Substitute it Now! List, SVHC).

All in all, five initiatives out of the twenty-four were selected, meeting all of our criteria. Two of the five initiatives were already included in CIPATOX-PE-2018 as they provided specific endocrine-disrupting effect data: EDS (10) and EDSP (9). The three newly included initiatives were “Database of Endocrine Disrupting chemicals and their Toxicity Profiles” (DEDUCT) (40, 41), a report by the Danish Center on Endocrine Disruptors (CeHoS) as a project contracted by the Danish Environmental Protection Agency (EPA) “List of Endocrine Disrupting Chemicals, Final report” (DANISH EPA) (42) and conclusions on pesticides from the EFSA “Peer review of the pesticide risk assessment” (EFSA) (43). The last volume included was published in August 2021 (44).

### 2.3 Endocrine target organs (ETO)

The endocrine target organs (ETO) retained in CIPATOX-PE- 2021, as for CIPATOX-PE-2018, were taken from the WHO report (45) Compared to CIPATOX-PE-2018, hematological/immunologic, hepatic, and neurological ETO have been added for the update as WHO describes immune and metabolic disorders (including hepatic) as well as neurobehavioral and developmental disorders as possibly related to endocrine-disrupting effects. In CIPATOX-PE-2018, for the EDS data, the endocrine-disrupting effects of these three ETO had been summarized in the category “other.” Although the data per ETO from EDS and EDSP previously entered in CIPATOX-PE-2018 have been rigorously preserved as such, it was necessary to reclassify the EDS data in the light of the three new ETO.

From the five selected initiatives, only the effects studied for humans or mammals have been considered in order to ensure that these data can be applied to human health in accordance with European regulations. All of the endocrine-disrupting effects on ETO studied in DEDUCT (18 ETO), the EFSA conclusion (4 ETO), and the DANISH EPA publication (6 ETO) were retained. For EDS and EDSP, the data for the 18 ETO and 6 ETO, respectively, already studied in CIPATOX-PE-2018 were kept (8).

All in all, CIPATOX-PE-2021 details endocrine-disrupting effects for 18 categories (Table 1) corresponding to ETO. These include those as follows:

**Table 1:** Summary of endocrine target organs (ETO) studied by each initiative.

	DEDUCT	EDS	EDSP	EFSA	DANISH EPA
Reproductive system					
Female reproductive organs	●	●	●		●
Male reproductive organs	●	●	●		●
Androgenic	●	●	●	●	●
Estrogens	●	●	●	●	●
Progesterone	●	●			
Offspring	●	●			●
Central (gonadotrophic axis)	●	●	●		
Reproductive system other	●	●		●	●
Metabolic					
Thyroid	●	●	●	●	
Parathyroids	●	●			
Adrenal glands	●	●			
Hypothalamic–pituitary axis	●	●			
Lipid metabolism	●	●			
Pancreas/Carbohydrate metabolism	●	●			
Hepatic	●	●			
Others	●	●			
Neurological	●	●			
Immunological/Hematological	●	●			

● Endocrine target organ studied. DEDUCT, Database of Endocrine Disrupting chemicals and their Toxicity profiles; EDSP, Endocrine Disruptor Screening Program Tier1; EDS, The Endocrine Disruptor Screening Program; EFSA, Peer review of the pesticide risk assessment of the active substance; DANISH EPA, List of Endocrine Disrupting Chemicals, Final report.

– For reproduction, eight ETO: female and male reproductive organs, estrogens, androgens, progesterone, gonadotropic axis, effects on offspring plus a category “reproduction other” (corresponding to reproductive effects that could not be classified in the other ETO).
– For metabolic pathways, seven ETO: thyroid gland, parathyroid glands, hepatic system, adrenal glands, hypothalamo-hypophyseal complex (excluding gonadotrophic axis), pancreatic islet and glucidic metabolism, lipidic metabolism.
– The neurological system.
– The hematological and immunologic system.
– A category called “other” is used for effects identified as endocrine disruptors by the initiatives but not classifiable by the method in the other existing ETO.

It is also important to note that for a given endocrine-disrupting effect, it can be classified in more than one ETO. For example, “increased prostate weight in offspring” is classified under the ETO “effect on offspring” as well as under “male reproductive organs.”

### 2.4 Extraction of data from different initiatives

The method employed to identify endocrine-disrupting effect evidence from each of the initiatives was as follows:

– For DEDUCT, DANISH EPA, and EFSA, the search was done by CAS numbers of PAS.
– For EDS and EDSP, the existing CIPATOX-PE-2018 method and data were used exclusively (8).

### 2.5 Building the endocrine-disrupting effect indicator

The level of evidence reported by each initiative for the identified endocrine-disrupting effects is quite diverse. As was done in CIPATOX-PE-2018, the construction of an endocrine-disrupting effect indicator was necessary to enable the synthesis of these data. From the level of evidence concluded by each initiative of an endocrine-disrupting effect, an endocrine-disrupting effect indicator was assigned. The level of evidence used depends on the level of evidence concluded by the initiative on the endocrine-disrupting effect and not on the type of study or scientific experimentation that led to their conclusions. Indeed, between initiatives, the type of study (e.g., *in vitro* and *in vivo*) and experimental model (e.g., murine and human) are not the same and do not necessarily lead to the same endocrine-disrupting effect conclusions. The indicator of effect thus allows the synthesis of the conclusions of the initiatives for each ETO and by PAS. The indicator has three levels, allowing to assign the level of concern of the endocrine-disrupting effect (Table 2):

**Table 2:** Endocrine disruptor (ED) effect indicator assigned based on endocrine-disrupting effect findings of initiatives.

	EDS	EDSP	DEDUCT	EFSA	DANISH EPA
Endocrine-disrupting effect of high concern	“Endocrine Disruptor” “CAT1” key study			“Is an endocrine disruptor” “Endocrine disruptor criteria are met”	“Endocrine Disruptor”
Endocrine-disrupting effect of concern	“Potential” “CAT 2” “CAT 1” non-key study	“Suspected” “yes”	“Potential”		“Suspected”
Unknown Not identified with endocrine-disrupting effect in the current state of knowledge	Insufficient data: “CAT 3b” No evidence for an endocrine-disrupting effect: “CAT 3a” Not known as Endocrine Disruptor according to the methodology for ETO studied by the initiative	“Suspected” “no”	No Endocrine Disruptor data identified in the literature by the method for the ETO studied by the initiative	“Data gap” “Need further investigation” “Is not an Endocrine Disruptor” “Does not meet Endocrine Disruptor criteria based on the available evidence” “Assess Endocrine Disruptor does not appear scientifically necessary”	No Endocrine Disruptor data identified in the methodology for the ETO studied by the initiative
PAS not studied	Not studied	Not studied	Not studied PAS studied but not retained according to their method (list of excluded substances not available)	Not studied	Not studied

ETO, Endocrine target organs; PAS, Phytopharmaceutical Active Substances; DEDUCT, Database of Endocrine Disrupting chemicals and their Toxicity profiles; EDSP, Endocrine Disruptor Screening Program Tier1; EDS, The Endocrine Disruptor Screening Program; EFSA, Peer review of the pesticide risk assessment of the active substance; DANISH EPA, List of Endocrine Disrupting Chemicals, Final report.

– ED effect of “high concern”
– ED effect of “concern”
– “unknown” effect for PAS not identified with endocrine-disrupting effect in the current state of knowledge.

The indicator was developed to reflect the best level of evidence concluded by the initiatives for each ETO and for a given PAS. Only three initiatives, EDS, EFSA, and DANISH EPA provide endocrine-disrupting effect conclusions to assign the highest indicator of “having an endocrine-disrupting effect of high concern.” The development of the endocrine-disrupting effect indicator is based on a “worst case” approach, so that if the conclusion of one of the initiatives allowed to assign endocrine-disrupting effect “of high concern,” the substance was classified as having an endocrine-disrupting effect “of high concern” even if another initiative gave a different endocrine-disrupting effect conclusion (e.g., “of concern”).

### 2.6 Analysis of data from CIPATOX-PE-2021

A descriptive statistical analysis of the CIPATOX-PE-2021 data was performed for all the PAS licensed in France from 1961 to 2021 according to CIPA. Descriptive analyses of the endocrine-disrupting effect indicator according to ETO were also performed. All analyses were performed using SAS software, version 9.4.

### 2.7 Data comparison between CIPATOX-PE-2018 and 2021

Data from CIPATOX-PE-2018 and CIPATOX-PE-2021 were compared to observe the impact of considering an ED-only mode of action. To do this, the two versions were compared for the same PAS (i.e., PAS registered before 2015). In order to allow comparison of the two versions, the CIPATOX-PE-2018 endocrine-disrupting concern level indicator was aligned with the update. Thus, in the first version of CIPATOX-PE in 2018, the pesticides studied were classified according to the data available in the following 4 categories: levels of “very high concern”, “medium concern”, “low concern” and “unknown”. For comparisons with the new version of CIPATOX-PE in 2021, the categories “medium concern” and “low concern” are grouped together under the designation “of concern”.

## 3 Results

### 3.1 CIPATOX-PE-2021

CIPATOX-PE-2021 includes 980 PAS, corresponding to the PAS licensed in France according to CIPA between 1961 and 2021, i.e., 71 more PAS than CIPATOX-PE-2018.

The initiatives have investigated between 3 and 152 PAS contained in the CIPATOX-PE-2021 database (Table 3).

**Table 3:** Summary by initiative of the number of phytopharmaceutical active substances for plant protection products (PAS) studied according to the three levels of the endocrine disruptor (ED) effect indicator.

	ED effect of high concern	ED effect of concern	Unknown endocrine-disrupting effect according to the current state of knowledge	Total number of PAS studied by the initiative
DEDUCT	NA	152	NA	152
EDS	42	34	59	135
EFSA	2	NA	43	45
EDSP	NA	21	23	44
DANISH EPA	2	1	NA	3

DEDUCT, Database of Endocrine Disrupting chemicals and their Toxicity profiles; EDSP, Endocrine Disruptor Screening Program Tier1; EDS, The Endocrine Disruptor Screening Program; EFSA, Peer review of the pesticide risk assessment of the active substance; DANISH EPA, Danish Center on Endocrine Disrupt 2017; NA, Non-Applicable.

Of the 980 PAS recorded in the CIPATOX-PE-2021 database, 739 PAS were not reviewed by any of the five initiatives and 241 PAS by at least one of the initiatives. The assessment came from a single initiative for 131 PAS (13.4%). In the study, 83 PAS (8.5%) were reviewed by two initiatives, 26 (2.6%) by three, and only one PAS by four initiatives.

Among the 241 PAS studied, 44 (18.2%) are identified according to the endocrine-disrupting effect indicator as having an endocrine-disrupting effect of “high concern,” 133 (5.2%) as “concern,” and 64 (26.6%) as “unknown” according to the current state of knowledge available and the methodology used.

**Figure 2:**
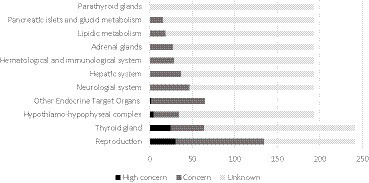
Number of phytopharmaceutical active substances (PAS) with an endocrine-disrupting effect (ED) indicator of “high concern,” “concern,” and “unknown” according to endocrine target organs (ETO).

The PAS with an endocrine-disrupting effect indicator of “high concern” involves 5 ETO (Figure 2). The ETO that is the target of the largest number of PAS with an endocrine-disrupting effect of “high concern” is the reproductive system with 31 PAS. This is followed by the thyroid with 25 PAS and the hypothalamic–pituitary axis (excluding the gonadotropic axis) with 5 PAS.

Finally, the “other endocrine target organs” and “neurological” ETO have 2 and 1 PAS, respectively, with an endocrine-disrupting effect of “high concern.”

All ETO are affected by one or more PAS with an endocrine-disrupting effect indicator of “concern.” In decreasing order, we find the reproductive system (104 PAS), the “other endocrine target organs” category (63 PAS), the neurological system (46 PAS), the thyroid gland (39 PAS), the hepatic system (37 PAS), the hypothalamic–pituitary axis (30 PAS), the hematological and immunologic system (29 PAS), the adrenal gland (28 PAS), lipid metabolism (19 PAS), carbohydrate metabolism and pancreas (16 PAS), and finally the parathyroids (1 PAS).

**Figure 3:**
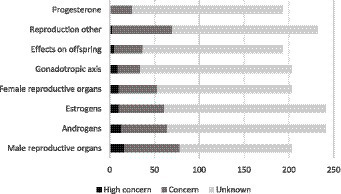
Number of phytopharmaceutical active substances (PAS) with an endocrine disruptor (ED) effect indicator of “high concern,” “concern,” and “unknown” according to the endocrine target organ “reproductive system”.

The “reproductive system” class has eight “ETO” subcategories, as shown in Figure 3. All of them are targets of PAS with an endocrine-disrupting effect indicator of “high concern,” ranging from 16 PAS for “male reproductive organs” to 1 PAS for the ETO “progesterone,” with an average of 9 PAS for all ETO combined. The top four most represented ETO are therefore “male reproductive organs,” “androgens,” “estrogens,” and “female reproductive organs.”

### 3.2 Comparison of data between CIPATOX-PE-2018 and 2021

Among the 909 PAS licensed between 1961 and 2014, 454 are studied by the CIPATOX-PE-2018 initiatives compared to 218 for CIPATOX-PE-2021. Among the PAS studied, the number of PAS with the PE effect indicator “high concern” for CIPATOX-PE-2018 and 2021 are 43 (9.5%) and 44 (20.2%), respectively. In addition, the number of PAS with the PE effect indicator “of concern” for CIPATOX-PE-2018 and 2021 are 298 (65.5%) and 131 (59.8%), respectively.

Thus, 42 PAS with an endocrine-disrupting effect of “high concern” are similarly classified in CIPATOX-PE-2018 and 2021, both identified by EDS. In this study, 2 new PAS are identified as having an endocrine-disrupting effect of “high concern” in the update: 1 PAS identified by the DANISH EPA and 1 PAS identified by EFSA. Both of these PAS were previously classified as having an endocrine-disrupting effect of “concern” in CIPATOX-PE-2018. Finally, a PAS was identified as having an endocrine-disrupting effect of “high concern” in CIPATOX-PE-2018 according to the CLP initiative but is now classified as a PAS not investigated for endocrine-disrupting effects in CIPATOX-PE-2021.

In this study, 28 PAS were not studied by the initiatives considered by CIPATOX-PE-2018 and are now studied by the CIPATOX-PE-2021 initiatives. Of these, 19 PAS are classified as “of concern” and 9 as “unknown effect.” On the other hand, 264 PAS are no longer considered in the update when data existed according to the criteria applied for CIPATOX-PE-2018, 1 PAS was considered in 2018 as having an effect of “high concern,” 170 PAS “of concern,” and 93 PAS identified “unknown” effect.

## 4 Discussion

Data from CIPATOX-PE-2018 were updated with the new definition and the new PAS licensed in France since 2015. A search and analysis of 24 initiatives of interest for the update was performed. Five international initiatives from governmental and non-governmental organizations, some of them recent, were selected, allowing the contribution of data for 18 ETO. This study provides an updated synthesis of the specific endocrine-disrupting effects of phytopharmaceutical active substances (PAS) that have been used in France over the past 60 years based on the compilation of data from existing initiatives and the current definition of endocrine-disrupting effects published by the European Union in 2018. The development of an endocrine-disrupting effect indicator, linked to the level of evidence reported by the initiatives on endocrine-disrupting effects, enables them to be synthesized and makes it easier to identify the PAS of concern.

However, this data compilation process has experienced some difficulties. Thus, the PAS listed in CIPA may have some synonyms. For example, the case of the PAS “formic aldehyde” and “formaldehyde” (CAS number: 50-0-0). They are in fact one and the same PAS but correspond to two distinct entities in CIPA, with their own data in terms of years of registration and types of use. Thus, it counts as two distinct PAS in CIPATOX-PE-2021 even though it is the same substance. In practice, in CIPATOX-PE-2021, the same CAS number can be associated with several PAS (e.g., “carbatene” and “metiram-zinc” with CAS number 9006-42-2), and a PAS can be associated with several CAS numbers (e.g., “copper” and its CAS numbers: 1332-65-6; 1317-39-1; 12069-69-1). Finally, some PAS do not have any CAS number listed (e.g., “lactic ferment”).

Only three out of the five initiatives provided conclusions on endocrine-disrupting effects with a level of evidence allowing for the assignment of the “high concern” endocrine-disrupting effect indicator: EDS, EFSA, and DANISH EPA. However, the latter two only investigated, respectively, 45 and 3 PAS of CIPATOXPE-2021. EDS studied 135 PAS. EDS data were already included in CIPATOX-PE-2018. Thus, in this update, the new initiatives contributing to the classification of PAS with a high level of evidence propose a low number of data compared to the number of PAS included in CIPATOX-PE-2021. Regarding the PAS identified as having an endocrine-disrupting effect indicator of “high concern,” EFSA identified 2 PAS. However, one of them was already identified by EDS as such. Concerning DANISH EPA, it also identified 2 PAS. Of these, one PAS was already identified by EDS as having an endocrine-disrupting effect indicator of “high concern.” Finally, the majority of PAS identified as having an endocrine-disrupting effect indicator of “high concern” is therefore from EDS data (42 PAS), yet these are identical with CIPATOX-PE-2018 as EDS data were already included.

Eventually, 64 PAS are classified as “unknown” based on the methodology and evidence available. This should not be interpreted as safe with respect to endocrine disruption but as the absence of a conclusion demonstrating an endocrine-disrupting effect due to documented scientific reasoning or insufficient data to conclude.

These “unknown” findings may evolve as more knowledge about endocrine-disrupting effects becomes available—as described above for the EDS and EDSP data—and as regulatory frameworks and standardized tests for endocrine disruptor assessment evolve.

The endocrine-disrupting effect indicator proposes a “worst case” strategy. For example, with this metric, a decision of an endocrine-disrupting effect by one initiative prevails over a decision of no endocrine-disrupting effect by another. This strategy is designed to identify PAS of concern with respect to the large number of licensed PAS, but it does not allow for the representation of alternative conclusions on endocrine-disrupting effects.

CIPATOX-PE-2018 identified data for 454 PAS compared to 218 PAS for CIPATOX-PE-2021. The number of PAS with the PE effect indicator of “high concern” was essentially identical between the two versions with 43 PAS in CIPATOX-PE-2018 and 44 in CIPATOX-PE-2021. Of these, 42 of the PAS were common to both versions as identified by EDS data.

CIPATOX-PE-2018 included toxicological data that were not only specific to endocrine-disrupting mode of action (JMPR, CLP, and EFSA’s “the Strategy for Identification of Endocrine Disruptors and Evaluation of their Cumulative Risks”). By taking these endocrine toxicity effects into account, more data were available for PAS. For CIPATOX-PE-2021, considering the new European definition required the selection of initiatives with specific endocrine-disrupting effect data. Thus, the current European regulation allows a specific and consensual identification of endocrine disruptors. This specific definition implies that the toxicological particularities of ED (e.g., low-dose, non-dose-dependent, and transgenerational effects) should be considered as they are not usually evaluated, thus allowing for their better identification. Nevertheless, the current European regulation appears to be more restrictive for the identification of endocrine-disrupting effects of PAS because the specific endocrine disruptor data available are still limited.

The assessment of endocrine-disrupting effects is challenging due to their particularities such as delayed, low-dose, non-dose related, transgenerational, species-dependent, and chronic and cumulative exposure to other toxicants (46). This increases the complexity of identifying and assessing the endocrine-disrupting effects of substances. An endocrine disruptor is a recent concept with mechanistic understanding and toxicological evaluations still limited. The “cocktail” effects are not regulated as toxicology tests are not based on an integrative approach to the effects of mixtures (46). In addition, substances used in the past have sometimes never been evaluated for endocrine-disrupting effects and may not be evaluated in the future as regulatory authorities such as EFSA give priority to the study of currently licensed or registered PAS. Thus, some PAS may never have endocrine disruptor test data due to lack of study, while endocrine-disrupting health effects may occur late, and some pesticides are persistent. The “transparency regulation” (47), in application since March 2021, is part of the reinforcement of the transparency of risk assessment in the food chain and should allow the public to access scientific studies and information submitted to EFSA by manufacturers. This information could allow the emergence of other non-governmental initiatives to identify endocrine-disrupting effects or to complete the data of existing ones. The European regulatory commitment to assess ED should allow the systematic and standardized reporting of ED data for each licensed PAS and thus reduce the high number of PAS for which no data are available in CIPATOX-PE-2021. In addition, following the update, on 16 May 2022, the EASIS (32) team published the online database EASIS 2.0. This new initiative in endocrine-disrupting effect identification using the WHO definition may provide new data. In view of the increasing availability of endocrine-disrupting effect data, it seems worthwhile to carry on with the update of CIPATOX-PE.

The study aimed to update CIPATOX-PE-2018 with the European regulation currently applicable and to allow the study of the PAS recently added to the CIPA database. The construction of endocrine-disrupting effect indicators, in relation to the level of evidence provided by the endocrine-disrupting effect initiatives, allows the synthesis of updated data and the detection of PAS of concern for endocrine-disrupting effects. CIPATOX PE-2021 thus allows providing information on the effects on health of old and banned pesticides as well as for newly licensed pesticides. This makes it possible to have information on health effects for current but also past exposures as the effects can occur delayed and the pesticides are persistent. However, scientific data are constantly evolving, and our update of molecules used in France over the last 60 years as well as data on their endocrine-disrupting effects ends in 2021. As a result, the figures produced may be underestimated.

CIPATOX-PE-2021 can be used to monitor populations of workers exposed to PAS in the context of epidemiological studies and to support the characterization of health effects in the context of occupational exposure. Finally, the implementation of a hazard class for endocrine disruptors as part of the European CLP regulation (in the same way as for carcinogenic, mutagenic, and reprotoxic substances) has been recently decided by the UE (48). Indeed, a modification of the European CLP regulation regarding endocrine disruptors was published on 21 March 2023 in the official journal. It creates two classes for substances with an established or suspected endocrine-disrupting effect. These new classifications are expected to be implemented by 2025 with a potential impact in terms of prevention (48).

## Data availability statement

Publicly available datasets were analyzed in this study. This data can be found in Refs. (9, 10, 41–44).

## Author contributions

CR: Data curation, Formal analysis, Investigation, Methodology, Software, Writing – original draft, Writing – review & editing, Conceptualization, Resources, Validation. AP: Conceptualization, Data curation, Investigation, Methodology, Project administration, Resources, Software, Supervision, Validation, Writing – original draft, Writing – review & editing, Formal analysis. EF: Data curation, Formal analysis, Investigation, Methodology, Resources, Software, Validation, Writing – review & editing, Conceptualization. CL: Resources, Validation, Writing – review & editing, Investigation. AS: Resources, Validation, Writing – review & editing, Methodology. JS: Conceptualization, Data curation, Formal analysis, Investigation, Methodology, Project administration, Resources, Software, Supervision, Validation, Writing – original draft, Writing – review & editing. BC: Conceptualization, Methodology, Project administration, Resources, Supervision, Validation, Writing – original draft, Writing – review & editing.
